# Risk of Maltreatment-Related Injury: A Cross-Sectional Study of Children under Five Years Old Admitted to Hospital with a Head or Neck Injury or Fracture

**DOI:** 10.1371/journal.pone.0046522

**Published:** 2012-10-31

**Authors:** Joseph Jonathan Lee, Arturo Gonzalez-Izquierdo, Ruth Gilbert

**Affiliations:** Institute of Child Health, University College London, London, United Kingdom; Alberta Research Centre for Health Evidence, University of Alberta, Canada

## Abstract

**Objectives:**

To determine the predictive value and sensitivity of demographic features and injuries (indicators) for maltreatment-related codes in hospital discharge records of children admitted with a head or neck injury or fracture.

**Methods:**

Study design: Population-based, cross sectional study. Setting: NHS hospitals in England. Subjects: Children under five years old admitted acutely to hospital with head or neck injury or fracture. Data source: Hospital Episodes Statistics, 1997 to 2009. Main outcome measure: Maltreatment-related injury admissions, defined by ICD10 codes, were used to calculate for each indicator (demographic feature and/or type of injury): i) the predictive value (proportion of injury admissions that were maltreatment-related); ii) sensitivity (proportion of all maltreatment-related injury admissions with the indicator).

**Results:**

Of 260,294 childhood admissions for fracture or head or neck injury, 3.2% (8,337) were maltreatment-related. With increasing age of the child, the predictive value for maltreatment-related injury declined but sensitivity increased. Half of the maltreatment-related admissions occurred in children older than one year, and 63% occurred in children with head injuries without fractures or intracranial injury.

**Conclusions:**

Highly predictive injuries accounted for very few maltreatment-related admissions. Protocols that focus on high-risk injuries may miss the majority of maltreated children.

## Introduction

Clinicians must have a low threshold for considering physical abuse or neglect in injured children as prompt intervention may reduce the risk of further harm. [1] Detection requires clinical experience, but most injured children presenting to hospital are seen by relatively inexperienced ‘frontline’ trainee paediatricians or emergency department staff. Their first-hand experience of maltreatment is limited because abuse or neglect account for less than one in every 100 emergency department attendances for injury. [2] The literature frequently cites certain features of injury, such as intracranial injury or fracture in a young child, as indicative of a high risk of maltreatment. Some hospitals use guidelines that recommend these features should trigger further investigation of possible child maltreatment; for example, by asking a paediatrician who is experienced in child protection to assess the child. [1], [3], [4] An advantage of such guidance is that it focuses the scarce resources of experienced staff on children at high risk of child maltreatment. A disadvantage is that clinicians may give less attention to children with injury characteristics that indicate a relatively low risk of child maltreatment. If most maltreated children present with low risk characteristics such guidance could result in more cases of child maltreatment being missed than if the recommendations were not followed.

Balancing the need for indicators (patient characteristics such as age or type of injury) to have high predictive value (i.e. a high proportion of injury admissions with the indicator are maltreatment-related), with high sensitivity (the indicator picks up a high proportion of truly maltreated children), is well recognised in the evaluation of screening tests. In this report, we use national data for injured children admitted to hospital in England to quantify the predictive value and sensitivity for child maltreatment of indicators based on age and type of injury.

## Methods

### Overview

We firstly took the perspective of a clinician faced with an injured child, and determined the predictive value of age and type of injury for maltreatment-related admission, which was defined by ICD10 codes taken from electronic discharge records. We also used a multivariable model take into account age, sex, and socio-economic status. Second, we took a broader view (such as when writing a clinical guideline), and determined the sensitivity of each of the indicators. This was done by determining the proportion of all maltreatment-related injury admissions detected by the indicator. Third, we considered a public health perspective and calculated the incidence of maltreatment-related injures for each type of injury indicator at each age, using Poisson regression. In order to take account of developmental differences, we stratified all analyses by developmental age bands.

### Population

We conducted a cross-sectional study of all acute hospital admissions to the NHS in England of children aged one week to five years using hospital administrative data for 1997 to 2009 (known as Hospital Episode Statistics (HES) http://www.hesonline.nhs.uk/). The study population was restricted to children with head or neck injury or fracture because these injuries are readily recognisable and therefore unlikely to be affected by miscoding. They also reflect the most frequent and serious injury presentations in young children and are associated with an increased risk of maltreatment. [4]–[8] Estimates of the denominator population of children resident in England at each year of age and calendar year, were extrapolated from census data provided by the Office of National Statistics. [9]

### Outcome

The primary outcome measure, maltreatment-related injury admission, was defined as the presence of any code from two exclusive clusters of ICD-10 codes recorded as a diagnostic code in the child's electronic discharge record (Table S1). [10], [11] The most specific cluster contained codes for maltreatment syndrome or assault. The second cluster contained codes that reflected investigation for undetermined intent or concerns about the child's adverse social circumstances. Both clusters of codes were used together to define the main outcome measure, maltreatment-related codes.

These maltreatment-related ICD-10 codes were selected to be consistent with ‘alert’ features listed in UK national guidance from NICE (National Institute of Clinical Excellence) that should prompt the clinician to ‘consider’ or ‘suspect’ child maltreatment and take further action. [1] These codes therefore reflect clinical concern about possible maltreatment but do not reflect a definitive diagnosis of abuse. They identify children who should be investigated further, and who require further assessment and information sharing. This classification reflects clinical reality where the cause of injury may not be known for certain until sometime after admission, if at all. To exclude these children from the analysis would bias estimates of predictive values, increasing estimates in the most suspicious injuries and decreasing them in less clear cut cases. To assess the robustness of our findings, we repeated analyses using the more specific set of codes for maltreatment syndrome or assault (Table S1, results in figures S1 and S2).

In a separate validation study in one hospital, both clusters of maltreatment-related codes were compared with clinician-entered text in electronic records. The codes were highly specific (90%, 20/22; personal communication, Gilbert), similar to findings from validation studies in the US and Australia. [12]–[14] Comparisons of rates and risk factors for these maltreatment-related codes in different countries have shown consistent results. [10], [11]

### Age and type of injury indicators

Previous studies have shown that an injured child's risk of maltreatment declines with age and is strongly related to development. [6], [8], [15] Types of injury also vary with developmental age. [1], [6] We therefore performed analyses separately for children admitted before six months of age (pre-mobile), between six to 12 months (mobility increasing), and between one to four years of age (mobile). Within these strata, we included indicators for age (one week to one month, one to three months and quarterly till 12 months, then each year of age).

We included indicators for types of injury that have been associated with a high risk of maltreatment in systematic reviews, for example, intracranial injury (ICI), skull fracture, long bone fracture, and thoracic or rib fractures. [4]–[8] Smaller groupings were avoided to prevent spurious findings due to multiple testing and small cell sizes. Because we restricted analyses to children with head or neck injury or fracture, the baseline category for comparison was children without other indicators: i.e. children with head injury without ICI or fracture. Such children could have any other form of head injury including bruising, laceration, incision and soft tissue injuries.

### Statistical analyses

All analyses were based on data for the entire 12-year period. The incidence of maltreatment-related injury admission and the injury grouping were stable over this time. [10] Each analysis was performed separately for the three age strata.

**Figure 1:**
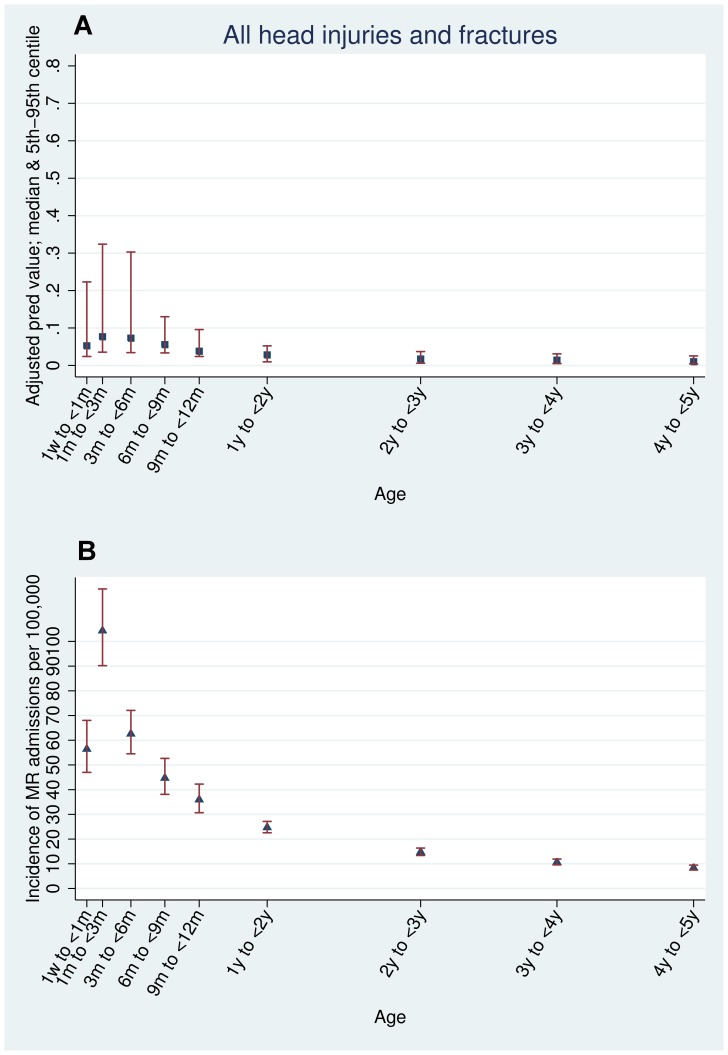
All injuries. Estimated predictive value and incidence of maltreatment-related admission by age (adjusted for sex, deprivation, and type of injury): all admissions for head or neck injury or fracture.

**Figure 2:**
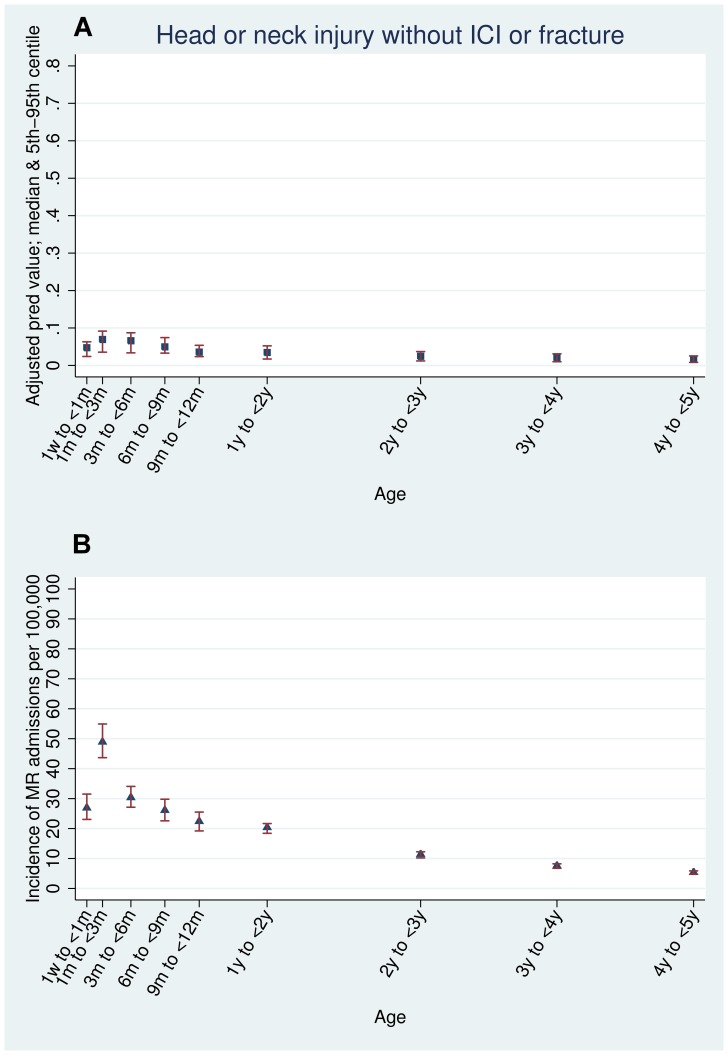
Head and neck injuries. Estimated predictive value and incidence of maltreatment-related admission by age (adjusted for sex, deprivation, and type of injury): all admissions for head or neck injury without intracranial injury or fracture.

**Figure 3:**
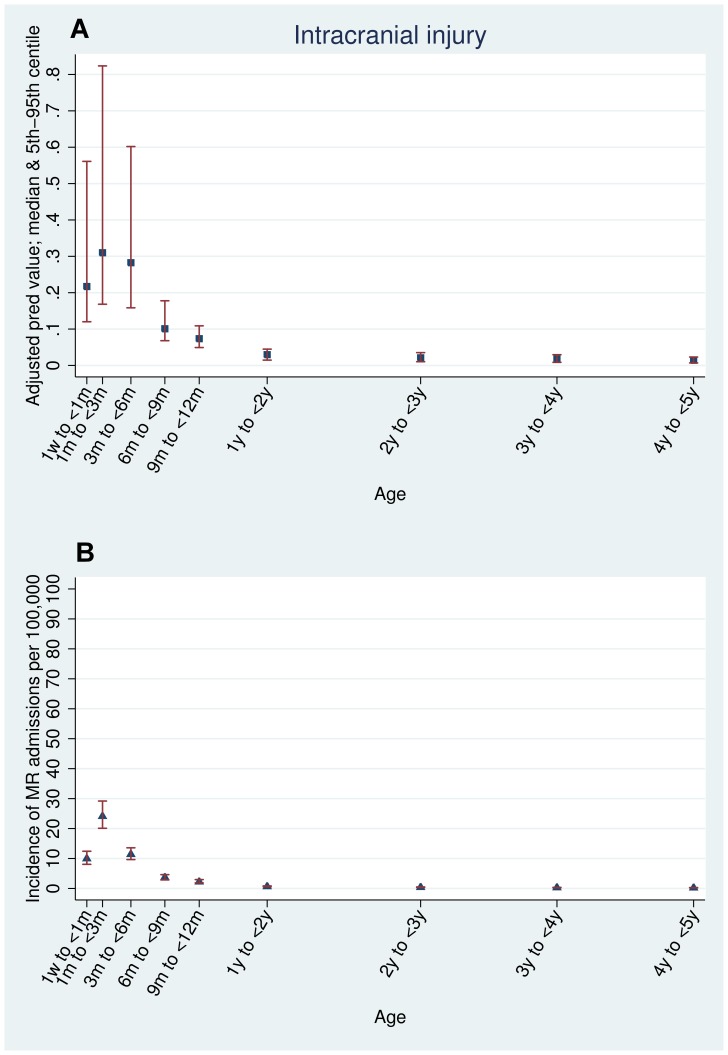
Intracranial injuries. Estimated predictive value and incidence of maltreatment-related admission by age (adjusted for sex, deprivation, and type of injury): all admissions for intracranial injury.

The crude predictive value was calculated as the proportion of all acute admissions with each type of injury that had maltreatment-related codes (Table 1). Adjusted estimates of the predictive value were calculated using logistic regression models with age, sex and quintile of multiple deprivation as *a priori* confounders for each age stratum. These models were used to assess the association between each type of injury and maltreatment-related codes by computing adjusted odds ratios (table 2). They were also used to predict the median probability (with 5^th^ to 95^th^ centiles) of maltreatment-related injury for each age and injury indicator (figures 1, 2, and 3).

**Table 1:** Characteristics of children admitted to hospital with head or neck injury or fracture in England 1997–2009.

*Characteristic*		*Number (%) with characteristic*	*Predictive Value (% MR)* *
**Age 1 week–6 months: All**		**27,128 (100)**	**9.9**
**Age**	1 w to <1 m	3,781 (13.9)	7.0
	1 m to <3 m	11,947 (44.0)	10.7
	3 m to <6 m	11,400 (42.0)	10.1
**Sex**	Female	12,524 (46.2)	9.3
	Male	14,604 (53.8)	10.5
**Deprivation quintile**	*Most deprived 1*	9,260 (34.1)	12.8
	2	5,918 (21.8)	10.9
	3	4,474 (16.5)	9.3
	4	3,847 (14.2)	6.3
	*Least deprived* 5	3,629 (13.4)	5.8
**All admissions aged 6–12 m:**		**27,198 (100)**	**5.4**
**Age**	6 m to <9 m	12,710 (46.7)	6.5
	9 m to <12 m	14,488 (53.3)	4.6
**Sex**	Female	12,362 (45.5)	5.3
	Male	14,836 (54.5)	5.6
**Deprivation quintile**	*Most deprived 1*	9,295 (34.2)	7.5
	2	5,912 (21.7)	5.4
	3	4,517 (16.6)	4.2
	4	3,694 (13.6)	3.6
	*Least deprived* 5	3,780 (13.9)	3.8
**All admissions aged 1–5 yrs:**		**205,968 (100)**	**2.0**
**Age**	1 to <2 yrs	57,297 (27.8)	3.1
	2 to <3 yrs	51,922 (25.2)	2.0
	3 to <4 yrs	47,189 (22.9)	1.6
	4 to <5 yrs	49,560 (24.1)	1.2
**Sex**	Female	85,275 (41.4)	1.9
	Male	120,693 (58.6)	2.1
**Deprivation quintile**	*Most deprived* 1	64,547 (31.3)	3.1
	2	42,148 (20.5)	2.2
	3	34,277 (16.6)	1.6
	4	31,460 (15.3)	1.2
	*Least deprived* 5	33,536 (16.3)	1.0

* %MR: The percentage of admissions maltreatment-related.

**Table 2:** Association between type of injury and maltreatment-related (MR) codes.

*Type of injury by age group*	*Number (%)*	*Predictive Value (%)* #	*MR incidence per 100,000 child years (95% CI)* ∧	*Sensitivity (% of all MR)* ∼	Adjusted odds ratio$ (95% CI)
**1 week to 6 months**					
**All injuries and fractures**	27128 (100)	9.9	76.2 (64.0–88.5)	32.3	
All head and neck injuries	24771 (91.3)	8.4	59.5 (51.6–68.9)	25	
Any fracture	6869 (25.3)	17.1	33.3 (27.8–40.1)	14.1	
**High risk injuries**					
Head or neck without ICI or fracture	19125 (70.5)	6.4	36.4 (32.3–41.0)	14.8	Baseline
ICI	1800 (6.6)	30.7	15.6 (13.1–18.8)	6.6	5.42 (4.81–6.10)**
Skull fracture	3337 (12.3)	9.2	8.7 (7.1–10.6)	3.7	0.93 (0.81–1.06)
Long bone	2217 (8.2)	28.8	18.1 (15.4–21.3)	7.7	4.75 (4.25–5.31)**
Thoracic	484 (1.8)	59.3	8.1 (6.6–10.1)	3.4	11.24 (9.15–13.81)**
**6 to 12 months**					
**All injuries and fractures**	27198 (100)	5.4	41.1 (32.1–50.1)	17.8	
All head and neck injuries	23430 (86.1)	5.1	31.8 (26.9–37.9)	14.3	
Any fracture	6910 (25.4)	7.8	14.3 (11.7–17.9)	6.4	
**High risk injuries**					
Head or neck without ICI or fracture	19459 (71.5)	4.5	24.3 (21.0–28.2)	10.5	Baseline
ICI	1134 (4.2)	9.7	3.0 (2.3–4.1))	1.3	2.15 (1.74–2.65)**
Skull fracture	2220 (8.2)	6.9	4.2 (3.2–5.4)	1.8	1.34 (1.12–1.60)*
Long bone	3672 (13.5)	8.7	8.7 (7.1–10.7)	3.8	1.99 (1.74–2.27)**
Thoracic	75 (0.3)	49.3	1.0 (0.7–1.5)	0.4	17.25 (10.72–27.77)**
**1 to 5 years**					
**All injuries and fractures**	205968 (100)	2	14.6 (9.3–20.0)	49.9	
All head and neck injuries	138427 (67.2)	2.6	11.4 (10.3–12.7)	42.7	
Any fracture	75606 (36.7)	1.2	2.9 (2.5–3.5)	10.6	
**High risk injuries**					
Head or neck without ICI or fracture	126320 (61.3)	2.5	10.6 (9.6–11.6)	37.8	Baseline
ICI	5344 (2.6)	2.2	0.4 (0.3–0.5)	1.4	0.85 (0.71–1.03)
Skull fracture	3662 (1.8)	3.2	0.4 (0.3–0.5)	1.4	1.22 (1.01–1.47)*
Long bone	63886 (31)	1	2.1 (1.8–2.4)	7.5	0.45 (0.42–0.50)**
Thoracic	149 (0.1)	12.8	0.06 (0.03–0.13)	0.2	6.58 (4.02–10.78)**

MR: Maltreatment-Related admissions, with codes for maltreatment, assault or adverse social circumstances.

# The percentage of admissions with each injury classified as maltreatment-related in each age group.

∧ Incidence of maltreatment-related admissions in children of each age group in England, by injury type.

∼ The contribution to the total of all 8,337 maltreatment-related admissions of children aged 1 w–5 y made by children in each age group with each injury, expressed as a percentage: the sensitivity of the injury.

$ Adjusted for injuries shown, socioeconomic status (deprivation quintiles), age and sex.

* p<0.05,

** p<0.001.

The sensitivity of each injury or age indicator was calculated as the proportion of all maltreatment-related admissions with the indicator. To reflect the public health burden of maltreatment-related injury, we estimated incidence rates of admission for each age-injury indicator, using Poisson regression offset by population estimates. The variance and mean were approximately equal and there was no evidence of over-dispersion. Admissions were not clustered by child as multiple admissions were rare.

Analyses were conducted in Stata 11 and R 2.13.0.

## Results

The 260,294 admissions with head or neck injury or fracture comprised 52.8% of all acute hospital admissions for injury in children aged one week to four completed years. Overall, 8,337 admissions (3.2%) had maltreatment-related codes, of which 45% were coded for maltreatment syndrome or assault. 94% of admissions were the first admission for injury and 2.4% of admissions had no code for the cause of injury.

### Predictive value

Table 1 shows that the predictive value for maltreatment-related injury admission given any type of head or neck injury or fracture declined with age from 9.9% in children less than six months old to 1.2% in children aged four years old. Results were similar for boys and girls. Predictive values for maltreatment-related admission increased steeply according to quintile of deprivation.

The types of injury indicator with the highest predictive value for maltreatment-related codes (table 2) were intracranial injury (30.7% in children under six months old) and thoracic fractures (rib, sternum or thoracic spine) in children aged less than one year (59.3% in children less than six months old, 49.3% in children six months to one year old). No injury indicators in children over six months old had a predictive value above 10% apart from thoracic injury. Head or neck injury without fracture or intracranial injury (ICI) had a low predictive value for maltreatment-related admission, even in children less than six months old (6.4%) (table 2). Table S2 shows the predictive value for each type of injury indicator for narrow age bands as plotted in figures 1, 2, and 3. The predictive values for all injuries peaked in children aged one to three months old and declined thereafter.

Among children under one year, adjusted odds ratios revealed that ICI was associated with a two- to five-fold increased risk of maltreatment-related injury admission compared with head or neck injury without ICI injury or fracture (table 2). However, there was no evidence of an association with ICI in children aged one to five years old. Thoracic fractures were associated with a six- to seventeen-fold increased risk of maltreatment-related admission, depending on age group (Table 2). Long bone fracture was associated with a four-fold increased risk of maltreatment-related admission in children under 12 months old, but in children aged one to five years was associated with a decreased risk, compared with head or neck injury without ICI or fracture. Skull fractures were not associated with maltreatment-related codes in children less than six months of age, but were weakly associated in children aged six months to one year.

### Sensitivity

Children aged from one to five years accounted for 49.9% of maltreatment-related admissions, while the highest risk age group (one week to six months) accounted for 32.3% (table 2). This was partly driven by the larger number of admissions in the age category one to five years compared with less than six months or six to twelve months.

**Figure 4:**
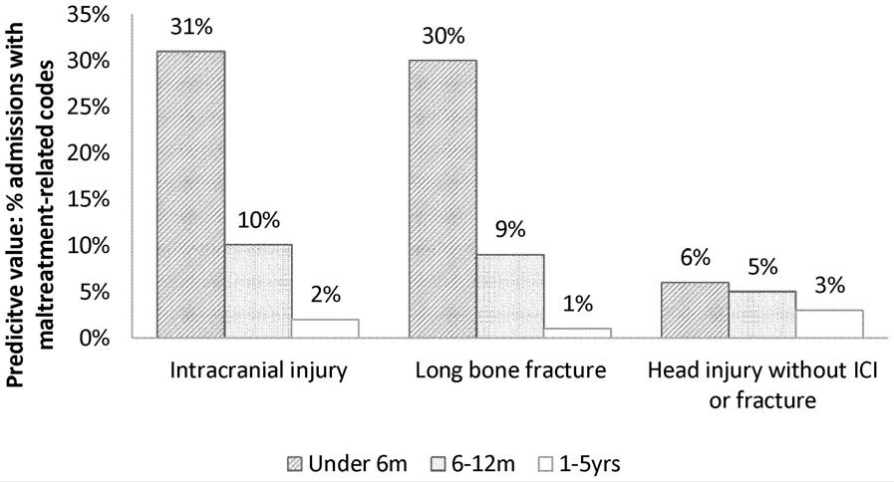
Predictive value of type of injury by age group.

**Figure 5:**
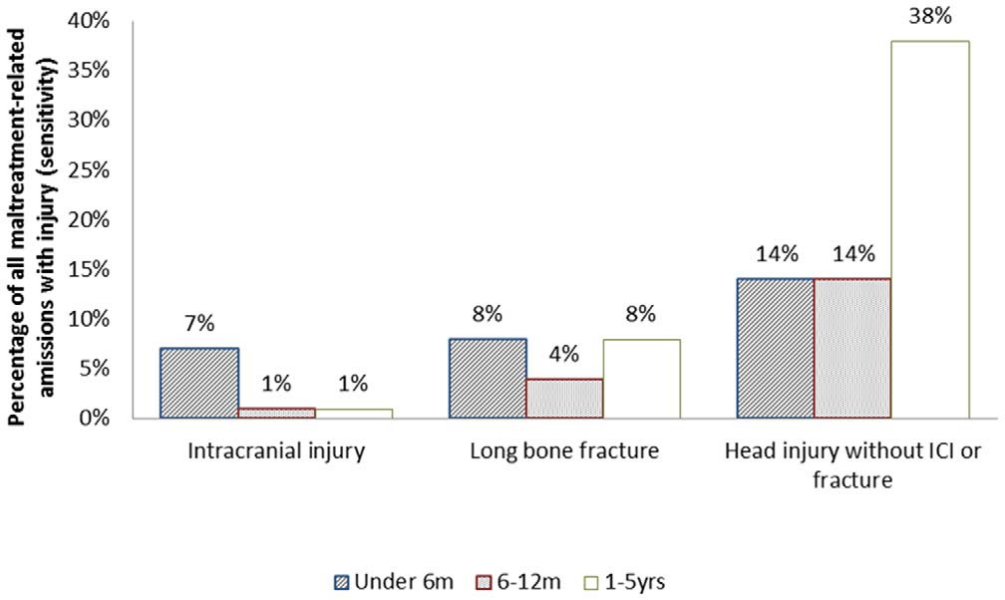
Sensitivity (% of all MR admissions represented by each age and injury group).

Head injury without ICI or fracture was the most sensitive indicator in all three age strata: when added together it accounted for 63.1% of maltreatment-related injury admissions in children under five years old. Although thoracic fractures were associated with the highest predictive value, they accounted for only four percent of maltreatment-related injury admissions (table 2). Predictive value and sensitivity are contrasted in figures 4 and 5. The age and injury indicators that were associated with a low predictive value for maltreatment-related codes accounted for the large majority of maltreatment-related injury admissions. This pattern was similar in the analyses restricted to the diagnostic codes for maltreatment syndrome or assault (figures S1 and S2).

### Incidence

The incidence of maltreatment-related admission head or neck injury or fracture was 58 per 100,000 children per year in children under one year of age, peaking at 104 per 100,000 between one and three months and then declining in older children (table S2, figures 1, 2, and 3). Children with head or neck injuries without ICI or fractures had the highest incidence of a single maltreatment-related injury, peaking at 49 per 100,000 (95% CI 43.7–54.9) in children aged one to three months. These injuries had a low predictive value for maltreatment, less than seven per cent at any age. In contrast, injuries with a high predictive value for maltreatment-related admission had low incidence rates. 61.6% of children with thoracic fractures in children aged one to three months had maltreatment-related admissions but the incidence was low (only 14 per 100,000 children) (table 2, figure 2, and table S2).

## Discussion

Half of the maltreatment-related admissions occurred in children aged one to five years old, particularly in children with types of injury that had a low predictive value for maltreatment. Few indicators for type of injury had a predictive value of more than 10%, rendering them of limited diagnostic use for clinicians deciding on the need for further assessment.

### Limitations

It is likely that maltreatment-related codes are under-recorded in hospital administrative data. Under-recording is also likely to vary with age and type of injury. [10], [13], [14], [16] The predictive values in our analyses should therefore be regarded as minimum estimates. Recording of maltreatment is likely to be best in the youngest children with high-risk injuries, as these groups are given most attention in guidelines. Our estimates of predictive value and incidence are therefore low-end estimates, particularly so for older children and those with injuries considered to be low risk.

Variation in under-recording with age differentially biases estimates of sensitivity. For example, our estimate that half of all maltreatment-related injuries occur in older children is likely to be an underestimate, as clinicians are less likely to recognise or record maltreatment in older age groups than in infants. A further source of error is that older children may be less likely than infants to be admitted when maltreatment is considered. Older children with recognised maltreatment-related injury are more likely than younger children to be discharged from the emergency department, with safeguarding follow-up in the community. [17], [18] These children could not be analysed in our database as emergency department attendances are not collated or coded in the same way as hospital admissions.

Detection bias increases estimates of predictive value because children not suspected of abuse are subject to different investigations than those who are under suspicion. For instance, only children suspected of maltreatment are subject to skeletal surveys. This phenomenon is most problematic for injuries that are unlikely to be apparent clinically, particularly rib fractures, and has been previously recognised by other studies. [5]

The inverse relationship between predictive value and sensitivity persisted when analyses were confined to the more specific cluster of ICD10 codes for maltreatment syndrome or assault (table S1, figures S1 and S2). We favoured the broader category of maltreatment-related injury admissions as we have previously shown evidence suggestive of diagnostic transfer between the specific cluster and the broader cluster of codes for undetermined intent or adverse social circumstances. These changes may have been due to a requirement for coders to record only definite or probable diagnoses from 2002 onwards. [10]

Our estimates of predictive value are lower than those seen in case series, but consistent with those from population-based studies. In a similar population-based study of inpatients in the USA, Leventhal *et al* found 24.8% of children aged less than three years with traumatic brain injuries or fractures were abused, and revealed the importance of fine age categories for the predictive value of maltreatment. [6] Case series report predictive values for maltreatment ranging from 30% to 67% for long bone fractures and 19% for head injuries. [3]–[5]

We found that predictive value, incidence and odds ratios were all highest in younger children, with a striking peak at one to three months of age (Figures 1, 2, and 3, table S2). We note that our findings are similar to those of Barr *et al*, who found that the peak incidence of maltreatment follows shortly after the peak of inconsolable crying in infants. [19] Additionally, a study of babies aged one to six months by Reijneveld *et al* found evidence for excessive crying as a trigger for potentially harmful carer behaviours such as smothering, slapping or shaking. [20]

### Implications

Our findings illustrate the potential harms of protocols that focus consideration of child maltreatment on the youngest children or children with high-risk injuries, as most maltreated children do not have these characteristics. To focus on incidence rates rather than predictive values does not solve this problem unless the size of the population at risk is also taken into account. Instead, all children presenting with injury should be assessed by clinicians with sufficient expertise to fully assess the child and their interaction with their parent for indicators of neglect, emotional abuse and physical maltreatment.

Our estimates of the predictive value of indicators for maltreatment-related injury are lower than reported in systematic reviews. The discrepancy is explained partly by under-recording of maltreatment in hospital administrative data reducing our estimates, and partly by selection biases in the case series and high-risk cohorts included in the reviews. Case series and high-risk cohorts are unlikely to include all accidental injuries, leading to artificially small denominators and overestimates of predictive value. [2], [10], [21] When used for judicial evidence, professionals should be aware that true predictive values are likely to lie between estimates from population-based administrative data and estimates reported in case series and high-risk cohorts.

### Conclusions

Guidance that focusses clinician attention on children at highest risk of maltreatment-related injury may falsely reassure clinicians and divert their attention from children with low-risk types of injury who make up the majority of maltreatment-related admissions.

## Supporting Information

Figure S1 **Repeated analysis using specific codes for maltreatment syndrome or assault: predictive value of type of injury by age group.**
(TIF)

Figure S2 **Repeated analysis using specific codes for maltreatment syndrome or assault: sensitivity (% of all MR admissions represented by each age and injury group).**
(TIF)

Table S1 Hierarchy of ICD 10 diagnostic codes* used to classify cause of injury related to child victimization.
(DOCX)

Table S2 **Maltreatment-related (MR) incidence, predictive value and sensitivity by finest age group and injury.**
(DOCX)
