# Supplementary material for: Risk of Maltreatment-Related Injury: A Cross-Sectional Study of Children under Five Years Old Admitted to Hospital with a Head or Neck Injury or Fracture
Source: PLoS One. 2012 Oct 31;7(10):e46522. doi: 10.1371/journal.pone.0046522 (PMC3485294; doi:10.1371/journal.pone.0046522)
Supplement: Table S1 — Hierarchy of ICD 10 diagnostic codes* used to classify cause of injury related to child victimization. (DOCX) [file pone.0046522.s003.docx]

|  | **Description** | **ICD-10 code** |
| --- | --- | --- |
|  | **1. Maltreatment** |  |
|  | Maltreatment syndromes | T74 |
|  | Perpetrator of neglect and other maltreatment syndromes | Y06, Y07 |
|  | **2. Assault** |  |
|  | Assault by bodily force | Y04, Y05 |
|  | Other types of assault | X85 - Y03, Y08 - Y09 |
|  | **3. Undetermined cause** |  |
|  | Events of undetermined intent | Y10 - Y34 |
|  | Blood alcohol and blood-drug tests | Z04.0 |
|  | Examination and observation following other inflicted injury | Z04.5 |
|  | Examination and observation for other reasons: request for expert evidence | Z04.8 |
|  | **4. Adverse social circumstances** |  |
|  | Neonatal withdrawal symptoms from maternal use of drugs of addiction | P96.1 |
|  | Problems related to social environment | Z60 |
|  | Problems related to negative life events in childhood | Z61 |
|  | Other problems related to upbringing | Z62 |
|  | Other problems related to primary support group | Z63 |
|  | Problems related to other legal circumstances | Z65.3 |
|  | Problems related to lifestyle (Except Z72.0 Tobacco use) | Z72 |
|  | Problems related to care-provider dependency | Z74 |
|  | Health supervision and care of foundling | Z76.1 |
|  | Health supervision and care of other healthy infant and child | Z76.2 |
|  | Family history of mental and behavioural disorders | Z81 |
|  | Personal history of other mental and behavioural disorders | Z86.5 |
|  | Personal history of other physical trauma | Z91.6 |
|  | Personal history of other specified risk-factors, not elsewhere classified | Z91.8 |

* In England (using HES) codes may be recorded in up to 20 diagnostic fields (14 before 2007 and 7 before 2002) for each hospital episode. Cause categories above are exclusive and arranged in a descending hierarchy.
