# Supplementary material for: Risk of Maltreatment-Related Injury: A Cross-Sectional Study of Children under Five Years Old Admitted to Hospital with a Head or Neck Injury or Fracture
Source: PLoS One. 2012 Oct 31;7(10):e46522. doi: 10.1371/journal.pone.0046522 (PMC3485294; doi:10.1371/journal.pone.0046522)
Supplement: Table S2 — Maltreatment-related (MR) incidence, predictive value and sensitivity by finest age group and injury. (DOCX) [file pone.0046522.s004.docx]

| **Injury and age** | **Incidence per 100,000 (95% CI)** | **% MR (Predictive value)** | **% of all MR (Sensitivity)** |
| --- | --- | --- | --- |
| All injuries <1m | 56.4(47.0-68.0) | 7.0 | 3.2 |
| All injuries 1-3m | 104.3(90.2-121.2) | 10.7 | 15.3 |
| All injuries 3-6m | 62.6(54.5-72.1) | 10.1 | 13.8 |
| All injuries 6-9m | 44.7(38.1-52.6) | 6.5 | 9.8 |
| All injuries 9-12m | 35.9(30.7-42.2) | 4.6 | 7.9 |
| All injuries 1-2y | 24.7(22.5-27.1) | 3.1 | 21.1 |
| All injuries 2-3y | 14.8(13.4-16.4) | 2.0 | 12.6 |
| All injuries 3-4y | 10.7(9.5-11.9) | 1.6 | 9.1 |
| All injuries 4<5y | 8.4(7.5-9.4) | 1.2 | 7.2 |
| Head and neck injuries <1m | 43.8(36.7-52.4) | 5.6 | 2.3 |
| Head and neck injuries 1-3m | 83.8(72.7-97.2) | 9.0 | 11.9 |
| Head and neck injuries 3-6m | 47.5(41.5-54.6) | 8.7 | 10.7 |
| Head and neck injuries 6-9m | 35.4(30.1-41.2) | 5.9 | 7.7 |
| Head and neck injuries 9-12m | 28.5(24.2-33.1) | 4.4 | 6.6 |
| Head and neck injuries 1-2y | 22.1(20.0-24.0) | 3.2 | 17.9 |
| Head and neck injuries 2-3y | 12.4(11.1-13.5) | 2.4 | 10.8 |
| Head and neck injuries 3-4y | 8.4(7.4-9.1) | 2.2 | 7.8 |
| Head and neck injuries 4<5y | 5.9(5.2-6.5) | 2.0 | 6.2 |
| Any fracture <1m | 25.1(20.3-31.3) | 11.0 | 1.4 |
| Any fracture 1-3m | 46.9(39-56.7) | 19.9 | 7.2 |
| Any fracture 3-6m | 26.4(22.3-31.4) | 16.5 | 5.5 |
| Any fracture 6-9m | 16.7(13.9-20.3) | 9.3 | 3.8 |
| Any fracture 9-12m | 12.1(10.2-14.8) | 6.2 | 2.6 |
| Any fracture 1-2y | 3.8(3.6-4.8) | 2.9 | 4.9 |
| Any fracture 2-3y | 3(2.9-3.7) | 1.2 | 2.5 |
| Any fracture 3-4y | 2.8(2.6-3.4) | 0.8 | 1.9 |
| Any fracture 4<5y | 2.8(2.6-3.3) | 0.4 | 1.3 |
| Intracranial injury <1m | 10(8.1-12.4) | 19.1 | 0.5 |
| Intracranial injury 1-3m | 24.1(20.1-29.2) | 34.7 | 3.5 |
| Intracranial injury 3-6m | 11.4(9.7-13.6) | 29.6 | 2.6 |
| Intracranial injury 6-9m | 3.7(2.9-4.6) | 13.9 | 1.0 |
| Intracranial injury 9-12m | 2.3(1.9-3.0) | 5.1 | 0.3 |
| Intracranial injury 1-2y | 0.7(0.6-0.9) | 3.0 | 0.6 |
| Intracranial injury 2-3y | 0.4(0.3-0.5) | 2.6 | 0.4 |
| Intracranial injury 3-4y | 0.3(0.2-0.4) | 1.8 | 0.3 |
| Intracranial injury 4<5y | 0.2(0.2-0.3) | 1.0 | 0.1 |
| Skull fracture <1m | 8.2(6.6-10.1) | 3.0 | 0.2 |
| Skull fracture 1-3m | 13(10.7-15.9) | 7.5 | 1.2 |
| Skull fracture 3-6m | 5.9(4.9-7.2) | 6.9 | 0.9 |
| Skull fracture 6-9m | 5.2(4.1-6.3) | 6.9 | 0.9 |
| Skull fracture 9-12m | 3.2(2.6-4.0) | 6.1 | 0.7 |
| Skull fracture 1-2y | 0.8(0.7-1.0) | 4.2 | 0.6 |
| Skull fracture 2-3y | 0.4(0.3-0.5) | 1.9 | 0.2 |
| Skull fracture 3-4y | 0.3(0.2-0.3) | 1.8 | 0.1 |
| Skull fracture 4<5y | 0.2(0.1-0.2) | 2.1 | 0.1 |
| Head injury, no fracture/ICI <1m | 26.9(23.1-31.5) | 4.8 | 1.5 |
| Head injury, no fracture/ICI 1-3m | 49(43.7-54.9) | 6.6 | 6.7 |
| Head injury, no fracture/ICI 3-6m | 30.4(27.1-34.1) | 6.8 | 6.6 |
| Head injury, no fracture/ICI 6-9m | 26.1(22.6-29.8) | 5.0 | 5.4 |
| Head injury, no fracture/ICI 9-12m | 22.4(19.2-25.5) | 4.0 | 5.1 |
| Head injury, no fracture/ICI 1-2y | 20.4(18.4-21.7) | 3.1 | 15.9 |
| Head injury, no fracture/ICI 2-3y | 11.5(10.2-12.2) | 2.4 | 9.7 |
| Head injury, no fracture/ICI 3-4y | 7.6(6.7-8.2) | 2.1 | 6.8 |
| Head injury, no fracture/ICI 4<5y | 5.4(4.7-5.9) | 1.9 | 5.4 |
| Long bone fracture <1m | 11.5(9.5-14.0) | 28.4 | 0.9 |
| Long bone fracture 1-3m | 23.9(20.2-28.4) | 34.7 | 3.7 |
| Long bone fracture 3-6m | 15.9(13.6-18.5) | 23.9 | 3.1 |
| Long bone fracture 6-9m | 9.9(8.2-11.6) | 10.9 | 2.3 |
| Long bone fracture 9-12m | 7.5(6.4-9.0) | 6.5 | 1.5 |
| Long bone fracture 1-2y | 2.3(2.1-2.6) | 2.9 | 3.6 |
| Long bone fracture 2-3y | 2.1(1.9-2.4) | 1.1 | 1.8 |
| Long bone fracture 3-4y | 2.1(1.9-2.4) | 0.6 | 1.2 |
| Long bone fracture 4<5y | 2.3(2-2.6) | 0.3 | 0.9 |
| Thoracic fracture <1m | 2.6(2.1-3.3) | 60.0 | 0.2 |
| Thoracic fracture 1-3m | 14.4(11.7-17.8) | 61.6 | 2.0 |
| Thoracic fracture 3-6m | 5.4(4.3-6.6) | 55.7 | 1.2 |
| Thoracic fracture 6-9m | 1.3(0.9-1.9) | 55.8 | 0.3 |
| Thoracic fracture 9-12m | 0.7(0.5-1) | 40.6 | 0.2 |
| Thoracic fracture 1-2y | 0.1(0.1-0.2) | 28.2 | 0.1 |
| Thoracic fracture 2-3y | 0.1(0-0.1) | 11.4 | 0.0 |
| Thoracic fracture 3-4y | 0.1(0-0.1) | 10.0 | 0.0 |
| Thoracic fracture 4<5y | 0(0-0.1) | 0.0 | 0.0 |
| This reference table shows, for each injury, the incidence, predictive value and sensitivity (percentage of all MR (maltreatment-related) admissions for which it accounts) at the finest age resolution that our data allows. Injuries with the highest predictive values have low incidence and sensitivity, being responsible for small proportions of the overall burden of maltreatment. | | | |
